# Supplementary material for: Curated and harmonised transcriptomics datasets of interstitial lung diseases
Source: Data Brief. 2025 Oct 14;63:112139. doi: 10.1016/j.dib.2025.112139 (PMC12581653; doi:10.1016/j.dib.2025.112139)
Supplement: Supplementary file 1 [file mmc1.zip › Supplementary_material/RNA-seq/fastQC reports/GSE180415/SRR15194698_2_fastqc.html]

SRR15194698\_2.fastq.gz FastQC Report 

FastQC Report

Wed 25 Oct 2023  
SRR15194698\_2.fastq.gz

## Summary

- Basic Statistics
- Per base sequence quality
- Per sequence quality scores
- Per base sequence content
- Per sequence GC content
- Per base N content
- Sequence Length Distribution
- Sequence Duplication Levels
- Overrepresented sequences
- Adapter Content

## Basic Statistics

| Measure | Value |
| --- | --- |
| Filename | SRR15194698\_2.fastq.gz |
| File type | Conventional base calls |
| Encoding | Sanger / Illumina 1.9 |
| Total Sequences | 40529791 |
| Sequences flagged as poor quality | 0 |
| Sequence length | 35-80 |
| %GC | 56 |

## Per base sequence quality

## Per sequence quality scores

## Per base sequence content

## Per sequence GC content

## Per base N content

## Sequence Length Distribution

## Sequence Duplication Levels

## Overrepresented sequences

| Sequence | Count | Percentage | Possible Source |
| --- | --- | --- | --- |
| GGGGGGGGGGGGGGGGGGGGGGGGGGGGGGGGGGGGGGGGGGGGGGGGGG | 89716 | 0.22135816096362304 | No Hit |
| GGTGGCGCGTGCCTGTAGTCCCAGCTACTCGGGAGGCTGAGGCTGGAGGA | 70146 | 0.17307269114711202 | No Hit |
| GGTGGCGCGTGCCTGTAGTCCCAGCTACTCGGGAGGCTGAGGTGGGAGGA | 64937 | 0.160220416631312 | No Hit |
| GATCAATCGCCCCCGGGGGTGCCTCCGGGCTCCTCGGGGTGCGCGGCTGG | 64596 | 0.15937906020783577 | No Hit |
| GCGGTGGCGCGTGCCTGTAGTCCCAGCTACTCGGGAGGCTGAGGCTGGAG | 59992 | 0.14801951483046139 | No Hit |
| GCGGTGGCGCGTGCCTGTAGTCCCAGCTACTCGGGAGGCTGAGGTGGGAG | 54476 | 0.13440977280144376 | No Hit |
| CCGGGCTCCTCGGGGTGCGCGGCTGGGGGTTCCCTCGCAGGGCCCGCCGG | 52845 | 0.13038557242991952 | No Hit |
| GTTCTGGGCTGTAGTGCGCTATGCCGATCGGGTGTCCGCACTAAGTTCGG | 51163 | 0.12623553869300733 | No Hit |
| CGGTGGCGCGTGCCTGTAGTCCCAGCTACTCGGGAGGCTGAGGCTGGAGG | 49016 | 0.12093820074226388 | No Hit |
| CGGTGGCGCGTGCCTGTAGTCCCAGCTACTCGGGAGGCTGAGGTGGGAGG | 45290 | 0.1117449631062741 | No Hit |

## Adapter Content

Produced by FastQC (version 0.11.7)
